# REHABILITATION APPROACH AFTER EARTHQUAKE DISASTER: A BRIEF REPORT FROM TURKEY

**DOI:** 10.2340/jrmcc.v7.34748

**Published:** 2024-04-22

**Authors:** Tuba Tülay KOCA, Duran TOPAK

**Affiliations:** 1 Department of Physical Medicine and Rehabilitation, Faculty of Medicine, Sütçü İmam University, Kahramanmaraş, Turke; 2 Department of Orthopedics and Traumatology, Faculty of Medicine, Sütçü İmam University, Kahramanmaraş, Turkey

**Keywords:** earthquake, disaster, musculoskeletal injury, rehabilitation, disability

## Abstract

Devastating earthquake disasters are experienced all over the world. On February 6, 2023, two major earthquakes with magnitudes of Mw 7.7 and 7.6, respectively, occurred centered in Kahramanmaraş, Turkey. It resulted in at least 50,783 deaths and more than 122,000 injuries according to official data. Defining the post-earthquake experiences and earthquake risk assessment well and identifying the deficiencies will guide the coordination, management, and planning of subsequent disasters. In this study, the rehabilitation approaches of earthquake victims with physical injuries in our rehabilitation center are emphasized and the situations that will be encountered in the immediate, intermediate, and long-term periods after the earthquake are summarized.

LAY ABSTRACT

At the beginning of 2023, Turkey experienced shocking earthquakes with magnitudes of mW 7.7 and 7.6. In this article, the patient profiles that can be seen in the immediate, intermediate, and long-term periods after the earthquake and the required rehabilitation approaches are explained. In the immediate post-earthquake period, we mostly encountered hemodynamic problems such as metabolic acidosis, shock, acute compartment syndrome, rhabdomyolysis, acute renal failure, and hypovolemia. The priority of the earthquake center should be to transport stable patients to appropriate places and provide first aid to unstable earthquake victims. In the intermediate period, hospital services gradually began to be provided in the city. During this period, we followed the profiles of patients who had surgical interventions, multiple extremity fractures, amputations, spinal cord injuries, peripheral nerve damage, and impaired functional ambulation. We included these patients in appropriate orthopedic, neurological, or amputee rehabilitation programs. In the long term, in addition to musculoskeletal injuries, earthquake victims suffered from head, neck, and back pain, dizziness, and balance disorders related to their accommodation. In addition to physical disability, psychological problems caused by the earthquake trauma were also observed.

Many countries around the world have experienced major earthquake disasters that have caused serious structural and human losses since ancient time. Countries living on major seismic fault lines have had to face this reality. Nations that learn from the experiences of major disasters and plan earthquake management well will cope better with the next disaster. Japan, Iran, Nepal, India, Pakistan, and Turkey can be considered as the countries that have experienced major earthquake disasters (1–5). Presenting reports based on the experiences of these earthquake-affected areas, conducting descriptive studies, revealing the deficiencies of rescue operation and analyzing the types of patients’ profiles, characteristics of injuries, and their treatment approaches will help preliminary preparation and planning for future earthquakes.

Turkey is a country with many seismic fault lines and has experienced major earthquakes throughout its history. For example, in 1544 and 1795 two major earthquakes caused serious destruction and damage in Kahramanmaraş province. On February 6, 2023, two major earthquakes with magnitudes of Mw 7.7 and 7.6 occurred, which centered in Kahramanmaraş and affected approximately 14 million people in 11 provinces. It resulted in at least 50,783 deaths and more than 122,000 injuries, according to the latest official data (6).

For this reason, in the post-earthquake period, it is necessary to determine the number of earthquake victims, their first aid and needs, and to prepare the necessary team and infrastructure materials (1, 2, 3, 7). Here, we especially defined patient profiles and needs in the post-earthquake period through the rehabilitation needs of earthquake victims with physical injuries. We believe that our experiences as a physiatrist an orthopedic surgeon and also as earthquake survivors will contribute to scientific disaster medicine.

## IMMEDIATE PERIOD

After the earthquake, in the first hours of infrastructural damage, health care structural damage, disruption of access to health care and transport, fuel crisis, etc. were assessed. Earthquake victims tried to cope with shelter and food problems. Victims were removed from the debris by the individual efforts of the public especially in the first 72 h and then by the efforts of trained technical rescue teams.

During the acute period after the earthquake, superficial injuries, open and closed fractures, open wounds due to high-energy trauma, compression-related compartment syndrome, crush injuries and compartment syndrome, vascular damage, shock, dislocation, and patients with multiple trauma are encountered in earthquake victims. In the acute period, some patients underwent orthopedic interventions such as amputation surgery, fasciotomy, tendon and soft tissue repair, neurovascular repair, debridement, fracture reduction, and fixation (4, 8). Some of the injuries were accompanied by infections. It was observed that the rescue time for these people in such a large earthquake varies depending on the work of the rescue teams.

While there were patients who stayed in the debris for 4 h after the earthquake, there were also patients who stayed in the wreckage for 10 days. For this reason and due to the inadequacy of the trained team, the timing of the surgery performed on these patients also varies (9, 10). After the earthquake, volunteer physicians and trained health staff from many other cities and countries came for helping to the earthquake-affected cities. It is more important that the humanitarian and material (food, clothing, shelter, medication, water, sanitation) aid received after the earthquake is organized rather than abundant. Aid arriving without any organization will increase the chaos in the city. For this reason, institutions should urgently create a coordination desk consisting of different members who coordinate and record internal and external aid.

After the earthquake, some hospital buildings in the city were also damaged and initial health services could not be provided and first interventions were given to patients in field hospitals. Difficulties related to access to health services can also be observed. In addition, systemic undesirable effects due to rhabdomyolysis, metabolic acidosis, acute renal failure, cold injury, hypovolemia, and hypoglycemia developed in patients who stayed in the wreckage for a long time. In addition, after being pulled from the wreckage, various problems may occur in the transfer process due to the inadequacy of the experienced team.

Since there were more applications than the capacity of the current services in the city, stable patients were referred to the nearest cities and unstable patients were treated in the city as much as possible.

## INTERMEDIATE PERIOD

In the intermediate period’ after the earthquake, we activated our outpatient and inpatient clinics approximately 2 months after the earthquake. A total of *N*=230 earthquake victims, 105 (45.7%) males and 125 (54.3%) females, aged between 1 and 79 years, applied to our Physical Medicine and Rehabilitation clinic. The youngest patient was a 16-month-old girl whose right ankle was trapped under debris for 9 h. The median time they stayed in the wreckage was 36 h (0–248 h).

Our patients mostly consisted of (1) lower extremity amputees, (2) those who developed loss of strength in the lower extremity due to vertebral fracture and spinal cord injuries, (3) with pelvic or extremity fractures, (4) with drop foot related to peroneal damage because of untreated compartment syndrome, (5) with brachial plexus damage in earthquakes or transfers, and (6) who have problems in limb use or mobilization due to these reasons. Soft tissue contracture development and reflex sympathetic dystrophy were also frequently observed. In patients with severe soft tissue loss, there were also limitations in adjacent joint movement. These patients were frequently accompanied by complaints of pain, so we regulated the pain medications of the patients. We applied peripheral nerve blocks for pain management.

**Fig. 1:**
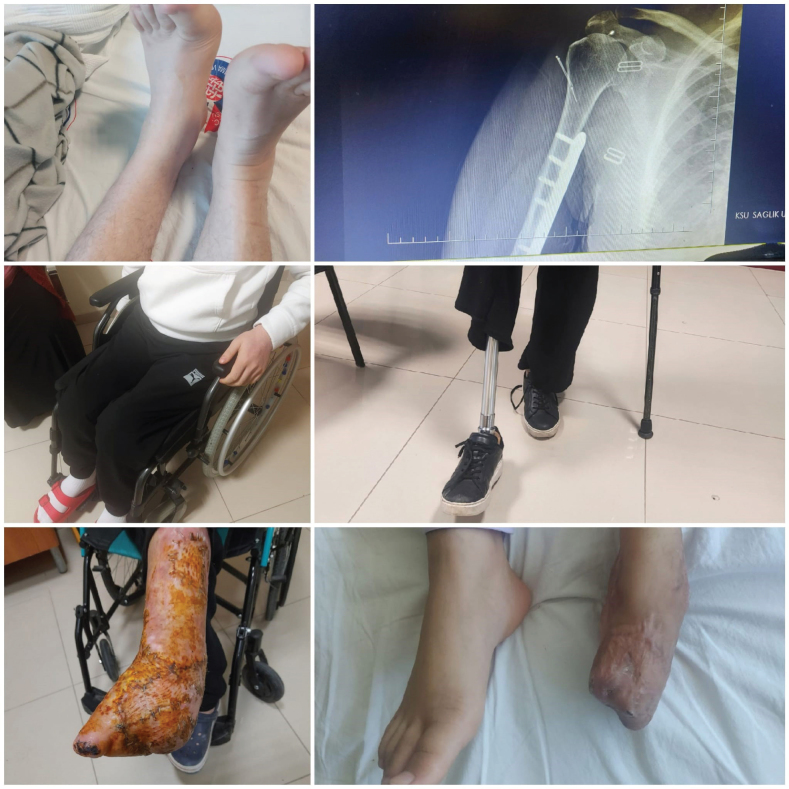
Some of our earthquake victims in rehabilitation process.

To ensure functional independent ambulation, we provided these patients with the necessary orthoses, prostheses, and assistive devices, and then initiated the neurological/orthopedic rehabilitation processes (Fig. 1). Some patients also had a history of staying in intensive care for a long time due to multiple organ failure or head trauma. Patients with chronic diseases before the earthquake experienced problems in the follow-up and treatment of these diseases and complications developed accordingly. An informed consent form was taken from the patients.

## LONG TERM PERIOD

In the long term, we were faced with the profile of an earthquake victim who had serious material and moral losses and needed psychological and physical support. In the same period, the city had turned into a “shelter city” and structural recovery and recovery efforts continue. Sleep problems, and head, neck, and back pain were frequently observed in earthquake victims who stayed in crowded environments and limited physical conditions in the container.

Apart from earthquake victims who suffered physical damage, we encountered post-earthquake dizziness syndrome, which is thought to be due to vertigo, vestibular dysfunction, autonomic dysfunction, postural balance disorder, or sensory problems, which negatively affect the daily lives of a significant number of individuals (5, 11, 12). Psychological, sleep problems, and ongoing physiological stress also contribute to this syndrome (13). Approximately 18,000 aftershocks hurt people’s recovery. We included these patients in a vertigo and balance, rehabilitation program.

Beyond physical disabilities, many of these patients had traces of mental health problems, anxiety, and depression after the earthquake, so we consulted the relevant clinics. Ongoing psychological and physical stress causes negative consequences for the individual in the short and long term, and these conditions affect the individual’s adaptation and success in rehabilitation. Children with physical injuries, especially those who have lost their parents, require special care and approach (14). Earthquake victims, children “earthquake orphans” (15), the elderly and women are special groups in need of help.

To summarize, millions of people around the world experience devastating earthquake disasters.

After these disasters, we encounter masses of people who have suffered serious losses, experienced psychological stress, and even have physical disabilities. Identifying the profiles of these individuals at an early stage and determining their needs in the short and long term should be among the rehabilitation goals. The rehabilitation approach to these people aims to provide functional recovery with early intervention, minimize complications, and physical and psychological disability, and reintegrate the person into society (3, 4, 7, 16, 17).
